# A hyperelastic model for simulating cells in flow

**DOI:** 10.1007/s10237-020-01397-2

**Published:** 2020-11-20

**Authors:** Sebastian J. Müller, Franziska Weigl, Carina Bezold, Christian Bächer, Krystyna Albrecht, Stephan Gekle

**Affiliations:** 1 Theoretical Physics VI, Biofluid Simulation and Modeling, University of Bayreuth, Universitätsstraße 30, Bayreuth, 95440 Germany; 2 Department of Functional Materials in Medicine and Dentistry and Bavarian Polymer Institute (BPI), University of Würzburg, Pleicherwall 2, Würzburg, 97070 Germany

**Keywords:** Hyperelasticity, Cell deformation, Mooney–Rivlin, Atomic force microscopy, Shear flow, Lattice-Boltzmann

## Abstract

In the emerging field of 3D bioprinting, cell damage due to large deformations is considered a main cause for cell death and loss of functionality inside the printed construct. Those deformations, in turn, strongly depend on the mechano-elastic response of the cell to the hydrodynamic stresses experienced during printing. In this work, we present a numerical model to simulate the deformation of biological cells in arbitrary three-dimensional flows. We consider cells as an elastic continuum according to the hyperelastic Mooney–Rivlin model. We then employ force calculations on a tetrahedralized volume mesh. To calibrate our model, we perform a series of FluidFM$^{{\textregistered }}$ compression experiments with REF52 cells demonstrating that all three parameters of the Mooney–Rivlin model are required for a good description of the experimental data at very large deformations up to 80%. In addition, we validate the model by comparing to previous AFM experiments on bovine endothelial cells and artificial hydrogel particles. To investigate cell deformation in flow, we incorporate our model into Lattice Boltzmann simulations via an Immersed-Boundary algorithm. In linear shear flows, our model shows excellent agreement with analytical calculations and previous simulation data.

**Electronic supplementary material:**

The online version of this article (10.1007/s10237-020-01397-2) contains supplementary material, which is available to authorized users.

## Introduction

The dynamic behavior of flowing cells is central to the functioning of organisms and forms the base for a variety of biomedical applications. Technological systems that make use of the elastic behavior of cells are, for example, cell sorting (Shen et al. 2019), real-time deformability cytometry (Otto et al. 2015; Fregin et al. 2019) or probing techniques for cytoskeletal mechanics (Kollmannsberger and Fabry 2011; Gonzalez-Cruz et al. 2012; Huber et al. 2013; Bongiorno et al. 2014; Fischer-Friedrich et al. 2014; Lange et al. 2015; Fischer-Friedrich et al. 2016; Nyberg et al. 2017; Lange et al. 2017; Kubitschke et al. 2017; Jaiswal et al. 2017; Mulla et al. 2019). In most, but not all, of these applications, cell deformations typically remain rather small. A specific example where large deformations become important is 3D bioprinting. Bioprinting is a technology which, analogously to common 3D printing, pushes a suspension of cells in highly viscous hydrogels—so-called bioink—through a fine nozzle to create three-dimensional tissue structures. A major challenge in this process lies in the control of large cell deformations and cell damage during printing. Those deformations arise from hydrodynamic stresses in the printer nozzle and ultimately affect the viability and functionality of the cells in the printed construct (Snyder et al. 2015; Blaeser et al. 2015; Zhao et al. 2015; Paxton et al. 2017; Müller et al. 2020). How exactly these hydrodynamic forces correlate with cell deformation, however, strongly depends on the elastic behavior of the cell and its interaction with the flowing liquid. Theoretical and computational modeling efforts in this area have thus far been restricted to pure fluid simulations without actually incorporating the cells (Khalil and Sun 2007; Aguado et al. 2012; Blaeser et al. 2015) or simple 2D geometries (Tirella et al. 2011; Li et al. 2015). The complexity of cell mechanics and the diversity of possible applications make theoretical modeling of cell mechanics in flow a challenge which, to start with, requires reliable experimental data for large cell deformations.

The most appropriate tool to measure cellular response at large deformations is atomic force microscopy (AFM) (Lulevich et al. 2003; Lulevich et al. 2006; Ladjal et al. 2009; Kiss 2011; Fischer-Friedrich et al. 2014; Hecht et al. 2015; Ghaemi et al. 2016; Sancho et al. 2017; Efremov et al. 2017; Ladjal et al. 2018; Chim et al. 2018). AFM cantilevers with pyramidal tips, colloidal probes, or flat geometries are used to indent or compress cells. Therefore, a common approach to characterize the elasticity of cells utilizes the Hertzian theory, which describes the contact between two linear elastic solids [(Johnson 2003), p. 90–104], but is limited to the range of small deformations (Dintwa et al. 2008). Experimental measurements with medium-to-large deformations typically show significant deviations from the Hertz prediction, e.g., for cells or hydrogel particles (Neubauer et al. 2019). Instead of linear elasticity, a suitable description of cell mechanics for bioprinting applications requires more advanced hyperelastic material properties. While for simple anucleate fluid-filled cells such as, e.g., red blood cells, theoretical models abound (Freund 2014; Závodszky et al. 2017; Mauer et al. 2018; Guckenberger et al. 2018; Kotsalos et al. 2019), the availability of models for cells including a complex cytoskeleton is rather limited. In axisymmetric geometries, Caille et al. (2002) and Mokbel et al. (2017) used an axisymmetric finite element model with neo-Hookean hyperelasticity to model AFM and microchannel experiments on biological cells. In shear flow, approximate analytical treatments are possible (Roscoe 1967; Gao and Hu 2009; Gao et al. 2011; Gao et al. 2012). Computationally, Gao and Hu (2009) carried out 2D simulations while in 3D Lykov et al. (2017) utilized a DPD technique based on a bead-spring model. Furthermore, Villone et al. (2014, 2015) presented an arbitrary Lagrangian-Eulerian approach for elastic particles in viscoelastic fluids. Finally, Rosti et al. (2018) and Saadat et al. (2018) considered viscoelastic and neo-Hookean finite element models, respectively, in shear flow.

In this work, we introduce and calibrate a computational model for fully three-dimensional simulations of cells in arbitrary flows. Our approach uses a Lattice-Boltzmann solver for the fluid and a direct force formulation for the elastic equations. In contrast to earlier works (Caille et al. 2002; Gao et al. 2011; Mokbel et al. 2017; Rosti et al. 2018; Saadat et al. 2018), our model uses a three-parameter Mooney–Rivlin elastic energy functional. To demonstrate the need for this more complex elastic model, we carry out extensive FluidFM$^{{\textregistered }}$ indentation experiments for REF52 (rat embryonic fibroblast) cells at large deformation up to 80% (Alexandrova et al. 2008). In addition, our model compares favorably with previous AFM experiments on bovine endothelial cells (Caille et al. 2002) as well as artificial hydrogel particles (Neubauer et al. 2019). Our model provides a much more realistic force-deformation behavior compared to the small-deformation Hertz approximation, but is still simple and fast enough to allow the simulation of dense cell suspensions in reasonable time. Particularly, our approach is less computationally demanding than conventional finite-element methods which usually require large matrix operations. Furthermore, it is easily extensible and allows, e.g., the inclusion of a cell nucleus by the choice of different elastic moduli for different parts of the volume.

We finally present simulations of our cell model in different flow scenarios using an Immersed-Boundary algorithm to couple our model with Lattice Boltzmann fluid calculations. In a plane Couette (linear shear) flow, we investigate the shear stress dependency of single cell deformation, which we compare to the average cell deformation in suspensions with higher volume fractions and show that our results in the neo-Hookean limit are in accordance with earlier elastic cell models (Gao et al. 2011; Rosti et al. 2018; Saadat et al. 2018).

## Theory

In general, hyperelastic models are used to describe materials that respond elastically to large deformations [(Bower 2010), p. 93]. Many cell types can be subjected to large reversible shape changes. This section provides a brief overview of the hyperelastic Mooney–Rivlin model implemented in this work.

The displacement of a point is given by

1 $$\begin{aligned} u_i = y_{i} - x_{i} \,, \end{aligned}$$

where $x_{i}$ ($i=1,2,3$) refers to the undeformed configuration (material frame) and $y_{i}$ to the deformed coordinates (spatial frame). We define the deformation gradient tensor and its inverse as [(Bower 2010), p. 14, 18]

2 $$\begin{aligned} \mathsf {F}_{ij} = \frac{\partial {y_{i}}}{\partial {x_{j}}} = \frac{\partial {u_i}}{\partial {x_{j}}} + \delta _{ij} \quad {\mathrm {and}} \quad \mathsf {F}_{ij}^{-1}= \frac{\partial {x_{i}}}{\partial {y_{j}}}\,. \end{aligned}$$

Together with the right Cauchy-Green deformation tensor, $\mathsf {C}= \mathsf {F}^{\intercal }\mathsf {F}$ (material description), we can define the following invariants which are needed for the strain energy density calculation below:

3 $$\begin{aligned} J= & {} \det \mathsf {F} \end{aligned}$$

4 $$\begin{aligned} I= & {} T_{\mathsf {C}} J^{-2/3} \end{aligned}$$

5 $$\begin{aligned} K= & {} \tfrac{1}{2}\left( T_{\mathsf {C}}^2 - T_{\mathsf {C}^2}\right) J^{-4/3} \end{aligned}$$

Here,

6 $$\begin{aligned} T_{\mathsf {C}} = {\mathrm {tr}}\,\mathsf {C} \quad {\mathrm {and}} \quad T_{\mathsf {C}^2} = {\mathrm {tr}}\,\left( \mathsf {C}^2\right) \end{aligned}$$

are the trace of the right Cauchy-Green deformation tensor and its square, respectively. The nonlinear strain energy density of the Mooney–Rivlin model is given by (Mooney 1940; Rivlin 1948)

7 $$\begin{aligned} U=\left[ \frac{\mu _1}{2} \left( I- 3\right) + \frac{\mu _2}{2} \left( K- 3\right) + \frac{\kappa }{2} \left( J-1\right) ^2\right] \,, \end{aligned}$$

where $\mu _1$, $\mu _2$, and $\kappa$ are material properties. They correspond—for consistency with linear elasticity in the range of small deformations—to the shear modulus $\mu =\mu _1+\mu _2$ and bulk modulus $\kappa$ of the material and are therefore related to the Young’s modulus $E$ and the Poisson ratio $\nu$ via [(Bower 2010), p. 74]

8 $$\begin{aligned} \mu = \frac{E}{2\left( 1+\nu \right) } \quad {\mathrm {and}} \quad \kappa = \frac{E}{3\left( 1-2\nu \right) } \,. \end{aligned}$$

Through the choice $\mu _2=0$ in (7), we recover the simpler and frequently used (Gao et al. 2011; Saadat et al. 2018) neo-Hookean strain energy density:

9 $$\begin{aligned} U_{\mathrm {NH}} =\left[ \frac{\mu }{2} \left( I- 3\right) + \frac{\kappa }{2} \left( J-1\right) ^2\right] \end{aligned}$$

As we show later, this can be a sufficient description for some cell types. To control the strength of the second term and quickly switch between neo-Hookean and Mooney–Rivlin strain energy density calculation, we introduce a factor $w\in \left[ 0,1\right]$ and set

10 $$\begin{aligned} \mu _1 = w\mu \quad {\mathrm {and}} \quad \mu _2 = (1-w)\mu \,\end{aligned}$$

such that $w=1$, which equals setting $\mu _2=0$ in (7), corresponds to the purely neo-Hookean description in (9), while $w<1$ increases the influence of the $\mu _2$-term and thus leads to a more pronounced strain hardening as shown in figure S-6 of the Supporting Information.

## Tetrahedralized cell model

**Fig. 1:**
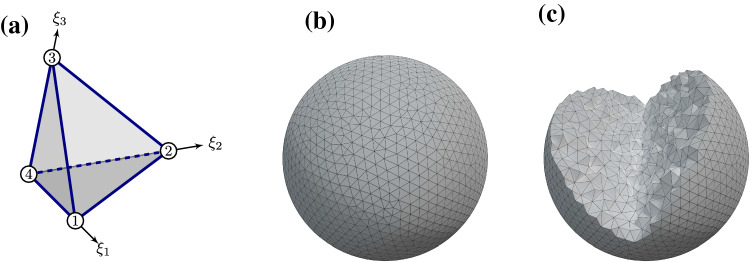
**a** The four noded tetrahedron as mesh element within a local dimensionless coordinate system $\left\{ {\xi }_1,{\xi }_2,{\xi }_3\right\}$. **b** The spherical cell model with its triangulated surface. **c** Its inner tetrahedralized mesh

In this section, we apply the hyperelastic theory of Sect. 2 to a tetrahedralized mesh as shown in Fig. 1.

### Calculation of elastic forces

We consider a mesh consisting of tetrahedral elements as depicted in Fig. 1. The superscript $\alpha$ refers to the four vertices of the tetrahedron. The elastic force acting on vertex $\alpha$ in direction *i* is obtained from (7) by differentiating the strain energy density $U$ with respect to the vertex displacement as

11 $$\begin{aligned} f_i^\alpha = - V_0 \frac{\partial {U}}{\partial {u_i^\alpha }} \,, \end{aligned}$$

where $V_0$ is the reference volume of the tetrahedron. In contrast to Saadat et al. (2018), the numerical calculation of the force in our model does not rely on the integration of the stress tensor, but on a differentiation where the calculation of all resulting terms involves only simple arithmetics. Applying the chain rule for differentiation yields:

12 $$\begin{aligned} f_i^\alpha= & {} -V_0 \left[ \left( \frac{\partial {U}}{\partial {I}} \frac{\partial {I}}{\partial {T_{\mathsf {C}}}} + \frac{\partial {U}}{\partial {K}} \frac{\partial {K}}{\partial {T_{\mathsf {C}}}}\right) \frac{\partial {T_{\mathsf {C}}}}{\partial {\mathsf {F}_{kl}}}\right. \nonumber \\&\left. +\left( \frac{\partial {U}}{\partial {I}} \frac{\partial {I}}{\partial {J}} + \frac{\partial {U}}{\partial {K}} \frac{\partial {K}}{\partial {J}} + \frac{\partial {U}}{\partial {J}} \right) \frac{\partial {J}}{\partial {\mathsf {F}_{kl}}}\right. \nonumber \\&\left. + \frac{\partial {U}}{\partial {K}} \frac{\partial {K}}{\partial {T_{\mathsf {C}^2}}} \frac{\partial {T_{\mathsf {C}^2}}}{\partial {\mathsf {F}_{kl}}}\right] \frac{\partial {\mathsf {F}_{kl}}}{\partial {u_i^\alpha }} \end{aligned}$$

The evaluation of (12) requires the calculation of the deformation gradient tensor $\mathsf {F}$, which is achieved by linear interpolation of the coordinates and displacements inside each tetrahedral mesh element as detailed in the next section. We note that our elastic force calculation is purely local making it straightforward to employ different elastic models in different regions of the cell and/or to combine it with elastic shell models. This flexibility can be used to describe, e.g., the cell nucleus (Caille et al. 2002) or an actin cortex (Bächer and Gekle 2019) surrounding the cell interior.

### Interpolation of the displacement field

Following standard methods, e.g., Bower (2010), we start by interpolating a point $x_{i}$ inside a single tetrahedron using the vertex positions $x_{i}^\alpha$ ($\alpha =1,2,3,4$). The interpolation uses an inscribed, dimensionless coordinate system, denoted by $\left( {\xi }_1,{\xi }_2,{\xi }_3\right)$ with $0\le {\xi }_i\le 1$^1^, as depicted in Fig. 1a. One vertex defines the origin while the remaining three indicate the coordinate axes. A set of shape functions, i.e., interpolation functions, ${N^{\alpha }}\left( {\xi }_1,{\xi }_2,{\xi }_3\right)$ is employed to interpolate positions inside the tetrahedron volume. An arbitrary point $x_{i}$ inside the element is interpolated as

13 $$\begin{aligned} x_{i} = \sum \limits _{\alpha =1}^{4} {N^{\alpha }}\left( {\xi }_1,{\xi }_2,{\xi }_3\right) x_{i}^\alpha \,, \end{aligned}$$

where the shape functions are defined as [(Bower 2010), p. 483]:

14 $$\begin{aligned} {N^{1}}\left( {\xi }_1,{\xi }_2,{\xi }_3\right)= & {} {\xi }_1 \end{aligned}$$

15 $$\begin{aligned} {N^{2}}\left( {\xi }_1,{\xi }_2,{\xi }_3\right)= & {} {\xi }_2 \end{aligned}$$

16 $$\begin{aligned} {N^{3}}\left( {\xi }_1,{\xi }_2,{\xi }_3\right)= & {} {\xi }_3 \end{aligned}$$

17 $$\begin{aligned} {N^{4}}\left( {\xi }_1,{\xi }_2,{\xi }_3\right)= & {} 1-{\xi }_1-{\xi }_2-{\xi }_3 \end{aligned}$$

According to (1), the displacement of vertex $\alpha$ in *i*-direction is given by

18 $$\begin{aligned} u_i^\alpha = y_{i}^\alpha - x_{i}^\alpha \,. \end{aligned}$$

Therefore similar to (13), the displacement at an arbitrary point in the volume can also be expressed in terms of the shape functions and the vertex displacements as

19 $$\begin{aligned} u_i = \sum \limits _{\alpha =1}^{4} {N^{\alpha }}\left( {\xi }_1,{\xi }_2,{\xi }_3\right) u_i^\alpha \,. \end{aligned}$$

The calculation of the deformation gradient tensor according to (2) requires the spatial derivative of the displacement:

20 $$\begin{aligned} \mathsf {F}_{ij} - \delta _{ij} = \frac{\partial {u_i}}{\partial {x_{j}}} = \frac{\partial {u_i}}{\partial {{\xi }_k}}\frac{\partial {{\xi }_k}}{\partial {x_{j}}} = \mathsf {A}_{ik} \mathsf {B}_{kj} \end{aligned}$$

By inserting (19) into (20) and evaluating the shape functions, the components of the matrix $\mathsf {A}$ are easily determined to be the difference of the displacements between the origin (vertex 4) and the remaining vertices 1, 2 and 3:

21 $$\begin{aligned} \mathsf {A}_{ik} = u_i^k - u_i^4 \end{aligned}$$

Note that due to the linear interpolation $\mathsf {A}_{ik}$ is constant inside a given tetrahedron. The matrix $\mathsf {B}=\mathsf {\mathsf {J}}^{-1}$ is the inverse of the Jacobian matrix, obtained similarly to (21) as

22 $$\begin{aligned} \mathsf {J}_{ik} = \frac{\partial {x_{i}}}{\partial {{\xi }_k}} = x_{i}^k - x_{i}^4 \,. \end{aligned}$$

Since $x_{i}$ refers to the reference coordinates, the calculation of the matrices $\mathsf {\mathsf {J}}$ and $\mathsf {B}$ has to be performed only once at the beginning of a simulation. With the interpolation of the displacement in each tetrahedron, we can write all derivatives occurring in (12), as listed in the following:

$$\begin{aligned} \frac{\partial {U}}{\partial {I}}= & {} \frac{\mu _1}{2} \qquad \qquad \frac{\partial {I}}{\partial {T_{\mathsf {C}}}} = J^{-\frac{2}{3}} \\ \frac{\partial {U}}{\partial {K}}= & {} \frac{\mu _2}{2} \qquad \qquad \frac{\partial {K}}{\partial {T_{\mathsf {C}}}} = T_{\mathsf {C}} J^{-\frac{4}{3}} \\ \frac{\partial {T_{\mathsf {C}}}}{\partial {\mathsf {F}_{il}}}= & {} 2 \mathsf {F}_{il}\qquad \qquad \frac{\partial {I}}{\partial {J}} = -\frac{2}{3} T_{\mathsf {C}} J^{-\frac{5}{3}} \\ \frac{\partial {K}}{\partial {J}}= & {} -\frac{2}{3} \left( T_{\mathsf {C}}^2 - T_{\mathsf {C}^2}\right) J^{-\frac{7}{3}}\qquad \frac{\partial {U}}{\partial {J}} = \kappa \left( J-1\right) \\ \frac{\partial {J}}{\partial {\mathsf {F}_{il}}}= & {} J\mathsf {F}_{li}^{-1}\qquad \qquad \frac{\partial {K}}{\partial {T_{\mathsf {C}^2}}} = -\tfrac{1}{2}J^{-\frac{4}{3}} \\ \frac{\partial {T_{\mathsf {C}^2}}}{\partial {\mathsf {F}_{il}}}= & {} 4 \mathsf {F}_{ik} \mathsf {C}_{kl}\qquad \qquad \frac{\partial {\mathsf {F}_{kl}}}{\partial {u_i^\alpha }} = \delta _{ki} \mathsf {B}_{ml} \left( \delta _{m \alpha } - \delta _{4\alpha } \right) \end{aligned}$$

### Taylor deformation parameter

As a measure for the cell deformation, we use the Taylor deformation parameter (Ramanujan and Pozrikidis 1998; Clausen and Aidun 2010; Guckenberger et al. 2016; Saadat et al. 2018)

23 $$\begin{aligned} D= \frac{a_3 - a_1}{a_3 + a_1} \,, \end{aligned}$$

where $a_1$ and $a_3$ are, respectively, the minor and major semi-axis of an ellipsoid corresponding to the inertia tensor of the cell. The Taylor deformation is a good measure for approximately elliptic cell deformations, as they occur in shear flow (cf. Sect. 6).

To calculate $D$, first the components of the inertia tensor

24 $$\begin{aligned} \mathsf {\Theta }_{ij} = \int \limits _V x_k x_k \delta _{ij} - x_i x_j {\mathrm {d}{V}} \,, \end{aligned}$$

where $\mathbf {x}$ is a vector inside the volume ${V}$, are calculated using our discretized cell with $N_{\mathrm {tet}}$ tetrahedra as

25 $$\begin{aligned} \mathsf {\Theta }_{ij} = \sum \limits _{l=1}^{N_{\mathrm {tet}}} V_l \left( r_k^l r_k^l \delta _{ij} - r_i^l r_j^l\right) \,. \end{aligned}$$

The vector $\mathbf {r}^{\,l}$ denotes the center of mass of the $l^{\mathrm {th}}$ tetrahedron and $V_l$ is its current volume. The eigenvalues $\theta _1> \theta _2 > \theta _3$ of $\mathsf {\Theta }$ can be used to fit the semi axes $a_1< a_2 < a_3$ of the corresponding ellipsoid:

26 $$\begin{aligned} a_1= & {} \frac{5}{2 M} \left( - \theta _1 + \theta _2 + \theta _3\right) \nonumber \\ a_2= & {} \frac{5}{2 M} \left( \theta _1 - \theta _2 + \theta _3\right) \nonumber \\ a_3= & {} \frac{5}{2 M} \left( \theta _1 + \theta _2 - \theta _3\right) \end{aligned}$$

The prefactor contains the mass $M$ of the ellipsoid (considering uniform mass density) and drops out in the calculation of $D$.

## Comparison of the numerical model to FluidFM$^{{\textregistered }}$ measurements on REF52 cells

In this section, we validate compression simulations of our cell model with FluidFM$^{{\textregistered }}$ compression experiments of REF52 cells stably expressing paxillin-YFP (Alexandrova et al. 2008). These experiments provide as an output the required force to produce a certain deformation of the cell, which can be directly compared to our model. We start with a detailed description of the experiments and show the suitability of our model to describe the elastic behavior of REF52 cells afterwards.

### FluidFM$^{{\textregistered }}$ indentation measurements

We perform a series of compression measurements of REF52 cells with a Flex FPM (Nanosurf GmbH, Germany) system that combines the AFM with the FluidFM$^{{\textregistered }}$ technology (Cytosurge AG, Switzerland). In contrast to conventional AFM techniques, FluidFM$^{{\textregistered }}$ uses flat cantilevers that possess a microchannel connected to a pressure system. By applying a suction pressure, cells can be aspirated and retained at the aperture of the cantilever’s tip. A more detailed description of the setup and its functionality is already reported in Sancho et al. (2017). All experiments are based on a cantilever with an aperture of 8${\mu}{\text{m}}$ diameter and a nominal spring constant of 2${\text{Nm}^{-1}}$. In order to measure the cellular deformation, a cell was sucked onto the tip and compressed between the cantilever and the substrate until a setpoint of 100nN was reached. Immediately before the experiment, the cells were detached by using Accutase (Sigma Aldrich) and were therefore in suspension at the time of indentation. In this way, it can be ensured that only a single cell is deformed during each measurement.

**Fig. 2:**
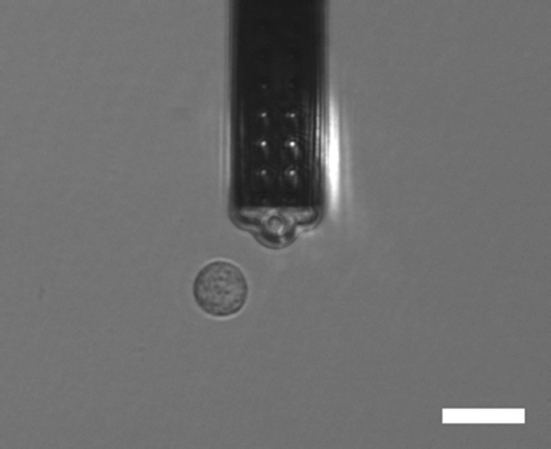
Example micrograph showing the FluidFM$^{{\textregistered }}$ cantilever and a cell viewed from the top. Scale bar is 30${\mu}{\text{m}}$

An example micrograph of the experiment before compression is shown in Fig. 2. Analogously to AFM, primary data in form of cantilever position (in ${m}$) and deflection (in ${\text{V}}$) has to be converted to force and deformation through the deflection sensitivity (in ${\text{m V}^{-1}}$) and the cantilevers’ spring constant. The cellular deformation further requires the determination of the contact point, which we choose as the cantilever position where the measured force starts to increase. The undeformed cell size is obtained as mean from a horizontal and vertical diameter measurement using the software imageJ.

### Simulation setup

**Fig. 3:**
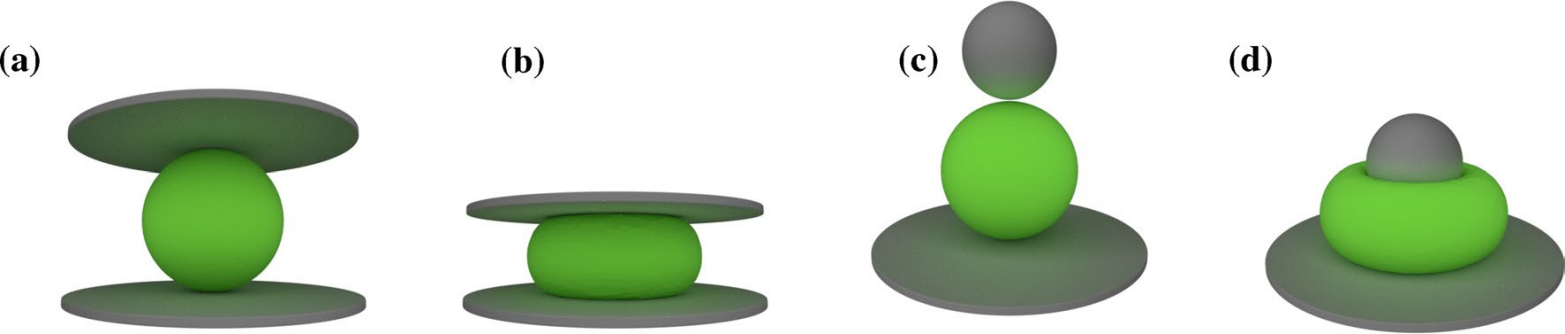
**a ** and **b** Cell compression simulations: The cell is compressed between a lower, resting, and an upper, moving, plate. **c** and **d** Colloidal probe cell indentation simulations: The cell rests on a plate, while being indented with a sphere

The experimental setup of the previous section is easily transferred and implemented for our cell model: The undeformed spherical cell rests on a fixed plate while a second plate approaches from above to compress the cell as depicted in Fig. 3a and b. In Sect. 5.2, we will also use a slightly modified version where a sphere indents the cell as shown in Fig. 3c and d. A repulsive force prevents the cell vertices from penetrating the plates or the spherical indenter. The elastic restoring forces (cf. Sect. 3) acting against this imposed compression are transmitted throughout the whole mesh, deforming the cell.

We use meshes consisting of 2000 to 5000 vertices and about 10000 to 30000 tetrahedra to build up a spherical structure. More details of the mesh and its generation (Sect. S-2.4) as well as the algorithm (Sect. S-3) are provided in the SI.

### Results

**Fig. 4:**
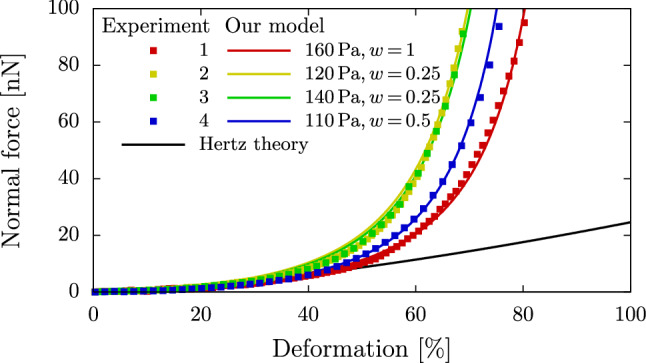
Our numerical model in comparison to our FluidFM$^{{\textregistered }}$ measurements on REF52 cells. The labels give the two fit parameters $E$ and $w$. We find Young’s moduli in the range of 110Pa to 160Pa. The Hertz theory is shown for a Young’s modulus of 180Pa

In our FluidFM$^{{\textregistered }}$ experiment series with REF52 cells, the cell radii lie between 7.1${\mu}{\text{m}}$ and 10.4${\mu}{\text{m}}$ with an overall average of 8.6±0.7${\mu}{\text{m}}$. In Fig. 4, we depict the force as function of the non-dimensionalized deformation, i.e., the absolute compression divided by the cell diameter. The experimental data curves share general characteristics: The force increases slowly in the range of small deformations up to roughly 40%, while a rapidly increasing force is observed for larger deformations. Although the variation of the cell radius in the different measurements is already taken into account in the deformation, the point of the force upturn differs significantly which indicates a certain variability in the elastic parameters of the individual cells.

We use the compression simulation setup as detailed in Sect. 4.2 to calculate force-deformation curves of our cell model. The Poisson ratio is chosen as $\nu =0.48$. In section S-2.7 of the Supporting Information, we show that variations of $\nu$ do not strongly affect the results. A best fit approach is used to determine the Young’s modulus and the ratio of shear moduli $w$ and leads to very good agreement between model prediction and experimental data as shown in Fig. 4 as well as section S-1 of the SI. While the general range of force values is controlled using the Young’s modulus, the Mooney–Rivlin ratio $w$ especially defines the point of the force upturn. We find Young’s moduli in the range 110Pa to 160Pa and $w=0.25$, 0.5, and 1. For very small deformations, our hyperelastic model produces the same results as would be expected from a linear elastic model according to the Hertz theory. See the SI (section S-2.5) for further details on the calculation of the force-deformation according to the Hertzian theory. For large deformations, the force rapidly increases due to its nonlinear character, showing strain-hardening behavior and huge deviations from the Hertz theory. Overall, we find an excellent match between simulation and our FluidFM$^{{\textregistered }}$ measurements with REF52 cells.

## Comparison of our numerical model to other micromechanical setups

In this section, we compare our simulations to axisymmetric calculations using the commercial software Abaqus and validate our cell model with further experimental data for bovine endothelial cells from (Caille et al. 2002) and very recent data for hydrogel particles from (Neubauer et al. 2019).

### Validation with axisymmetric simulations

To validate our model numerically, we compare our simulated force—deformation curves to calculations using the commercial software Abaqus (Smith 2009) (version 6.14).

**Fig. 5:**
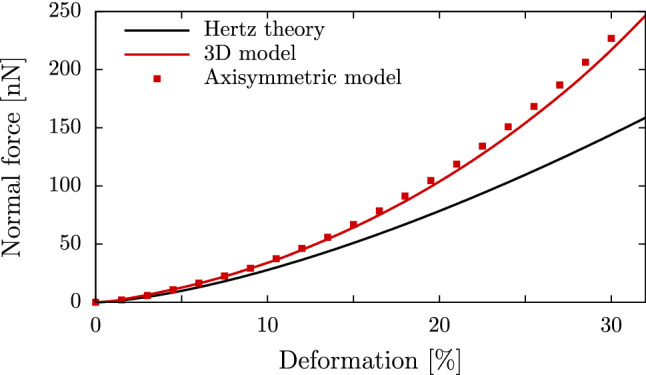
Comparison of force-deformation curves obtained from our model (red line) with the linear elastic Hertz theory (black line) and the two-dimensional simulation with Abaqus (red squares), showing good agreement between our three-dimensional and the axisymmetric model

In Abaqus, we use a rotationally symmetric setup consisting of a two-dimensional semicircle, which is compressed between two planes, similar to our simulation setup in Sect. 4.2 and the finite element model utilized in (Caille et al. 2002). The semicircle has a radius $r={15}{\mu }m$, a Young’s modulus of $E={2.25}{kPa}$ and a Poisson ratio of $\nu =0.48$. We choose a triangular mesh and the built-in implementation of the hyperelastic neo-Hookean model. In Fig. 5, we see very good agreement between the results of the two different numerical methods.

### Validation with AFM experiments

To compare with the AFM experiments of Caille et al. (2002), we simulate a cell with radius 15${\mu}{\text{m}}$ using the setup of Sect. 4.2. For the hydrogel particle indentation (Neubauer et al. 2019) we use the setup depicted in Fig. 3c and d with a particle radius of 40${\mu}{\text{m}}$ and a radius of the colloidal probe of 26.5${\mu}{\text{m}}$. The Poisson ratio is chosen as 0.48 in all simulations and the Young’s modulus is determined using a best fit to the experimental data points. Since the neo-Hookean description appears to be sufficient for these data sets, we further set $w=1$.

**Fig. 6:**
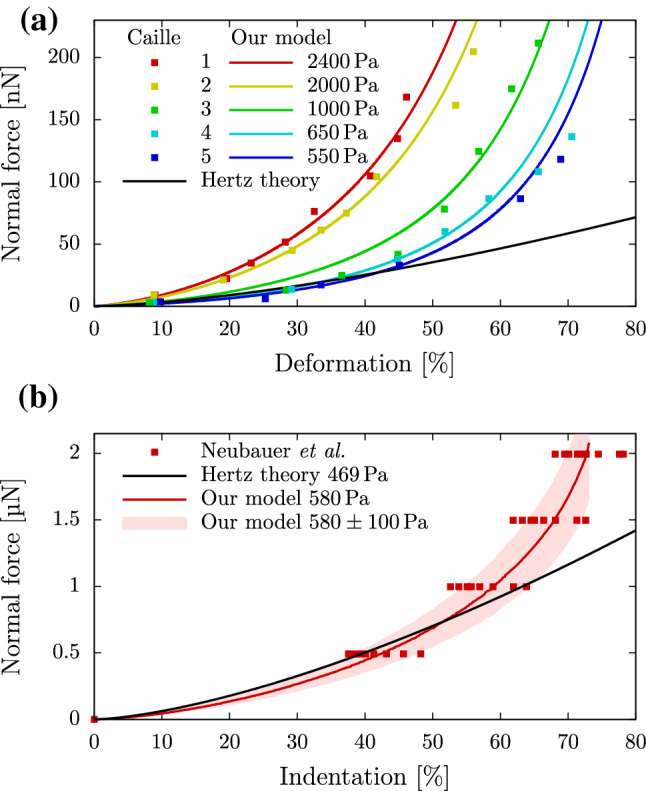
**a** Our numerical model in comparison to experimental measurements of bovine endothelial cells from (Caille et al. 2002). The black line depicts the prediction of the Hertz theory for a Young’s modulus of 1000Pa. **b** Our numerical model in comparison to experimental measurements of hydrogel particles from (Neubauer et al. 2019). The indicated range corresponds to the experimentally found range of ± 100Pa for the Young’s modulus according to the depicted Hertz model

In Fig. 6a, we show the experimental data for suspended, round, bovine endothelial cells of five separate measurements from (Caille et al. 2002) together with the prediction of the Hertz theory for a Young’s modulus of 1000Pa. Fitting our data with Young’s moduli in the range of 550Pa to 2400Pa, we find good agreement between our calculations and the experimental data. We note that Caille et al. (2002) observed similarly good agreement for their axisymmetric incompressible neo-Hookean FEM simulations which, however, cannot be coupled to external flows in contrast to the approach presented here. The same procedure is applied to the colloidal probe indentation data of hydrogel particles from (Neubauer et al. 2019), showing in Fig. 6b the experimental data and the prediction of the Hertz theory from (Neubauer et al. 2019). We find excellent agreement between our model calculations for Young’s moduli in the range of ${580}\pm {100}$Pa and the experimental data. For both systems, Fig. 6 shows large deviations between the Hertzian theory and the experimental data for medium-to-large deformations. Our model provides a significant improvement in this range.

## Application in shear flow

**Fig. 7:**
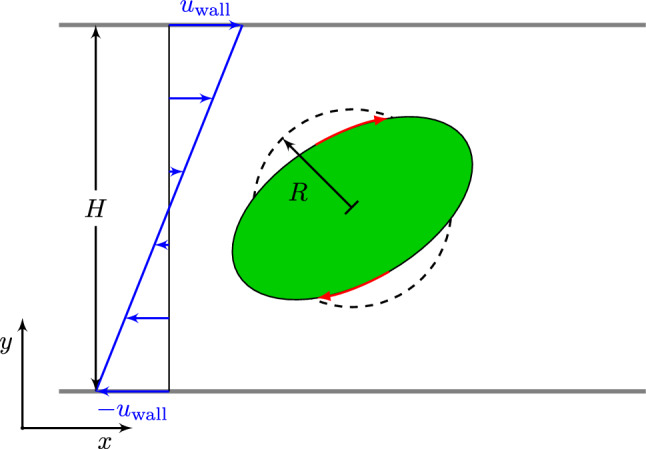
Schematic of the single cell in shear flow. The cell sits in the center of the box and shows an approximately elliptic deformation as well as tank-treading, i.e., a rotation of the membrane around the steady shape in the *x*-*y*-plane

We now apply our model to study the behavior of cells in a plane Couette (linear shear) flow setup and compare the steady cell deformation to other numerical and analytical cell models of Gao et al. (2011), Rosti et al. (2018) and Saadat et al. (2018). A sketch of the simulation setup is shown in Fig. 7. For simplicity, we choose $w=1$ to reduce the Mooney–Rivlin description (7) to two free parameters $\mu$ and $\kappa$ (or $E$ and $\nu$), obtaining a compressible neo-Hookean form. We use the Lattice Boltzmann implementation of the open source software package ESPResSo (Limbach et al. 2006; Roehm and Arnold 2012). Coupling between fluid and cell is achieved via the immersed-boundary algorithm (Devendran and Peskin 2012; Saadat et al. 2018) which we implemented into ESPResSo (Bächer et al. 2017; Bächer and Gekle 2019). We note here that, in contrast to Saadat et al. (2018), we do not subtract the fluid stress within the particle interior. This leads to a small viscous response of the cell material in addition to its elasticity. To obtain (approximately) the limit of a purely elastic particle, we exploit a recently developed method by Lehmann et al. (2020) to discriminate between the cell interior and exterior during the simulation. Using this technique, we can tune the ratio between inner and outer viscosity $\lambda$ with $\lambda \rightarrow 0$ representing a purely elastic particle. For simplicity, we will nevertheless set $\lambda =1$ in the following, except where otherwise noted. Details of the method are provided in the SI (section S-4.1). As measure for the deformation, we investigate the Taylor parameter $D$ (23) of our initially spherical cell model in shear flow at different shear rates $\dot{\gamma }$.

### Single cell simulation

The first simulation setup, a single cell in infinite shear flow, is realized by choosing a simulation box of the dimensions $10\times 15\times 5$ ($x \times y \times z$) in units of the cell radius. The infinite shear flow is approximated by applying a tangential velocity $u_{\mathrm {wall}}$ on the *x*-*z*-planes at $y=0$ in negative and at $y=15$ in positive *x*-direction, as depicted in Fig. 7. The tangential wall velocity is calculated using the distance *H* of the parallel planes and the constant shear rate $\dot{\gamma }$ via

27 $$\begin{aligned} u_{\mathrm {wall}} = \tfrac{1}{2}H \dot{\gamma }\,. \end{aligned}$$

**Fig. 8:**
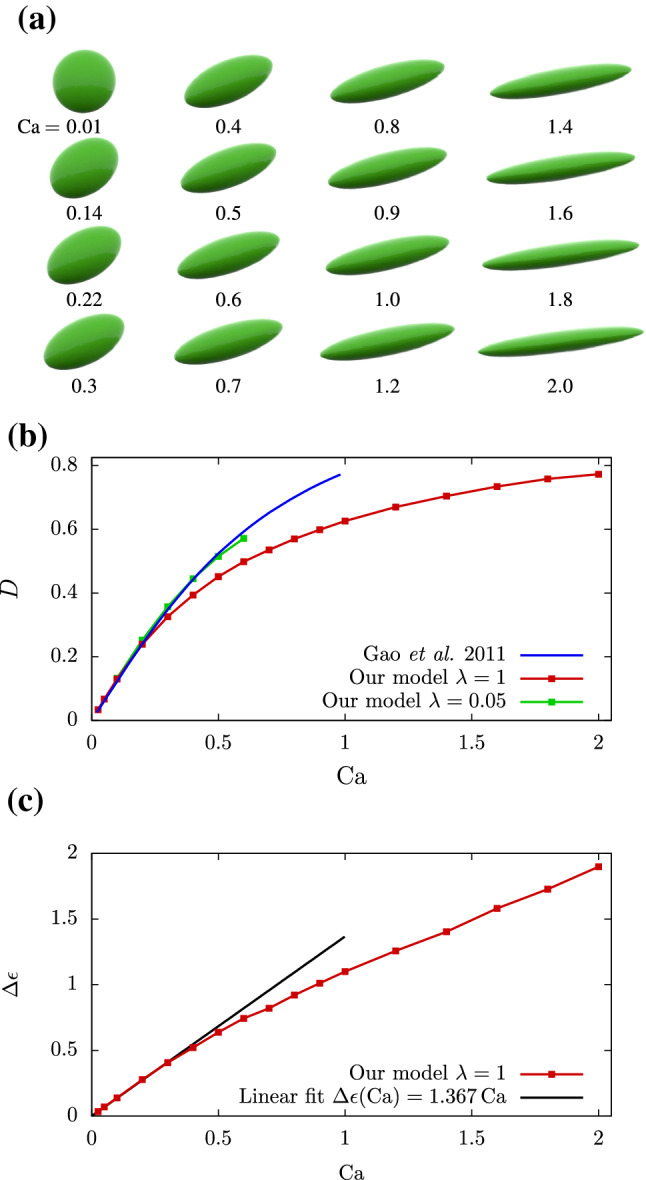
**a** Converged shapes of a single cell in a $10\times 15\times 5$ ($x \times y \times z$) simulation box (in units of the cell radius) with a shear flow in *x*-direction as function of the capillary number ${\mathrm {Ca}}$. **b** Comparison of our model predictions for a single cell in shear flow to the analytical 3D calculations in Fig. 7 of Gao et al. (2011) in the range of ${\mathrm {Ca}}\in \left[ 0.01,2.0\right]$. **c** The relative stretch $\Delta \epsilon$ of our cell model as function of the capillary number ${\mathrm {Ca}}$. A linear behavior is found for small capillary numbers up to ${\mathrm {Ca}}=0.3$, while increasing stress is required for larger deformations due to the strain-hardening quality of the neo-Hookean model. Lines are a guide to the eye

The box is periodic in *x* and *z*. A single cell is placed at the center of the simulation box corresponding to a volume fraction of $\phi =0.0003$. We choose the following parameters: fluid mass density $\varrho = 10^{3}kg m^{-3}$, dynamic viscosity $\eta =10^{-3}$Pas, and shear rate $\dot{\gamma }=4s^{-1}$. The capillary number is defined by (Gao and Hu 2009)

28 $$\begin{aligned} {\mathrm {Ca}}= \frac{\eta \dot{\gamma }}{\mu } \,, \end{aligned}$$

and is used to set the shear modulus $\mu$ of our cell relative to the fluid shear stress $\eta \dot{\gamma }$. Simulation snapshots of the steady-state deformation of a single cell in shear flow are depicted in dependency of the capillary number in Fig. 8a. We compare the Taylor deformation parameter $D$ to previous approximate analytical calculations of Gao et al. (2011) for a three-dimensional elastic solid in infinite shear flow in Fig. 8b and see reasonable agreement for our standard case of $\lambda =1$. Reducing the inner viscosity by setting $\lambda =0.05$, i.e., close to the limit of a purely elastic solid, the agreement is nearly perfect. Finally, we demonstrate that the elastic particle exhibits a tank-treading motion in section S-4.2.

A possibly even more intuitive way to measure cell deformation is the net strain of the cell which we define as

29 $$\begin{aligned} \Delta \epsilon = \frac{\left( d_{\mathrm {max}}-d_\mathrm {ref}\right) }{d_{\mathrm {ref}}} \,. \end{aligned}$$

It describes the relative stretching of the cell using the maximum elongation $d_{\mathrm {max}}$, i.e., the maximum distance of two cell vertices, and its reference diameter $d_{\mathrm {ref}}=2R$. A strain of $\Delta \epsilon ={1}$ thus corresponds to an elongation of the cell by an additional 100% of its original size. In Fig. 8c, we depict the $\Delta \epsilon$ as function of ${\mathrm {Ca}}$. For small capillary numbers, i.e., small shear stresses, a linear stress-strain dependency is observed. Above ${\mathrm {Ca}}\approx 0.3$, the strain-hardening, nonlinear behavior of the neo-Hookean model can be seen. By stretching the cell up to 280% of its initial size, this plot demonstrates again the capability of our model to smoothly treat large deformations.

### Multiple cell simulations

The second simulation setup, implemented to investigate the multiple particle aspect of our model, consists of 4 (8) cells in a $5\times 8\times 4$ simulation box (in units of the cell radius), corresponding to a volume fraction of $\phi =0.11$ ($\phi =0.22$) occupied by cells. The cells are inserted at random initial positions in the box and the flow parameters are the same as in the first setup (cf. Sect. 6.1).

**Fig. 9:**
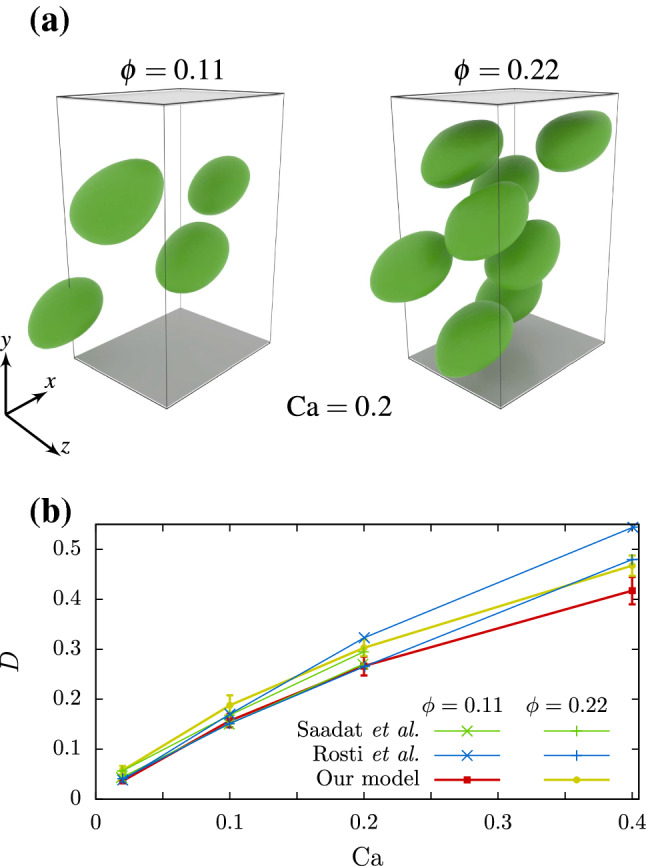
**a** Multiple cells in a $5\times 8\times 4$ ($x \times y \times z$) simulation box (in units of the cell radius) with a confined shear flow in *x*-direction for a capillary number of ${\mathrm {Ca}}=0.2$ and 4 cells corresponding to a volume fraction of $\phi =0.11$, and 8 cells corresponding to $\phi =0.22$. **b** Averaged deformation of multiple cell simulations with $\phi =0.11$ and $\phi =0.22$ in comparison to data from Fig. 3 of Rosti et al. (2018) and Fig. 13 of Saadat et al. (2018)

Figure 9a shows simulation snapshots of the cells in suspensions with volume fraction $\phi =0.11$ and $\phi =0.22$ for ${\mathrm {Ca}}=0.2$. The Taylor deformation of the suspensions, depicted in Fig. 9b, is calculated as an average over all cells and over time after an initial transient timespan. We find good agreement when comparing the averaged cell deformation in suspension with Rosti et al. (2018), Saadat et al. (2018).

## Conclusion

We presented a simple but accurate numerical model for cells and other microscopic particles for the use in computational fluid-particle dynamics simulations.

The elastic behavior of the cells is modeled by applying Mooney–Rivlin strain energy calculations on a uniformly tetrahedralized spherical mesh. We performed a series of FluidFM$^{{\textregistered }}$ compression experiments with REF52 cells as an example for cells used in bioprinting processes and found excellent agreement between our numerical model and the measurements if all three parameters of the Mooney–Rivlin model are used. In addition, we showed that the model compares very favorably to force versus deformation data from previous AFM compression experiments on bovine endothelial cells (Caille et al. 2002) as well as colloidal probe AFM indentation of artificial hydrogel particles (Neubauer et al. 2019). At large deformations, a clear improvement compared to Hertzian contact theory has been observed.

By coupling our model to Lattice Boltzmann fluid calculations via the Immersed-Boundary method, the cell deformation in linear shear flow as function of the capillary number was found in good agreement with analytical calculations by Gao et al. (2011) on isolated cells as well as previous simulations of neo-Hookean and viscoelastic solids (Rosti et al. 2018; Saadat et al. 2018) at various volume fractions.

The presented method together with the precise determination of model parameters by FluidFM$^{{\textregistered }}$ /AFM experiments may provide an improved set of tools to predict cell deformation— and ultimately cell viability—in strong hydrodynamic flows as occurring, e.g., in bioprinting applications.

## Electronic supplementary material

Below is the link to the electronic supplementary material.

Supplementary file1 (PDF 836 kb)

## Footnotes

1 (Bower 2010), p. 481, 483] erroneously states a range of $-1\le {\xi }_i\le 1$ for the tetrahedral element.
