# Supplementary material for: A hyperelastic model for simulating cells in flow
Source: Biomech Model Mechanobiol. 2020 Nov 20;20(2):509–20. doi: 10.1007/s10237-020-01397-2 (PMC7979664; doi:10.1007/s10237-020-01397-2)
Supplement: Supplementary file 1 — Supplementary file1 (PDF 836 kb) [file 10237_2020_1397_MOESM1_ESM.pdf]

SUPPLEMENTARY MATERIAL FOR THE MANUSCRIPT

A hyperelastic model for simulating cells in flow

Sebastian J. Müller[1], Franziska Weigl[2], Carina Bezold[1], Christian Bächer[1], Krystyna Albrecht[2], and Stephan Gekle[1]

[1]Theoretical Physics VI, Biofluid Simulation and Modeling, University of Bayreuth, Universitätsstraße 30 Germany,
[2]Department of Functional Materials in Medicine and Dentistry and Bavarian Polymer Institute (BPI), University of Würzburg, Pleicherwall 2, 97070 Würzburg, Germany
Author to whom correspondence should be addressed:
stephan.gekle@uni-bayreuth.de

S-1 Supplementary Material for the cell experiments

Additional force-deformation curves for our FluidFM® measurements on REF52 cells are shown in figure S-1. Compared to the curves depicted in the manuscript in figure 4, these measurements show an earlier upturn of the force. Thus, our model overestimates the force necessary for a small deformation of the cell and slightly underestimates the force for larger deformations. Nevertheless, all measurements fit in the simulated range of $E = 220 \pm 100\,\mathrm{Pa}$ for $w = 0.25$ and an averaged cell radius of $8.6(7)\,\mu\mathrm{m}$, as figure S-1 shows. The cell radii and Young's moduli for all measurements are listed in table S-1.

Table S-1 Measured cell radii R and fitted Young's moduli E and w for our FluidFM® experiments.

Number	1	2	3	4	5	6	7	8	9
R [μm]	7.1	9.2	8.3	8.0	9.5	9.1	8.4	9.4	8.3
E [Pa]	160	190	220	170	210	290	210	220	125
w	1	0.25	0.25	0.5	0.25	0.25	0.25	0.25	0.25

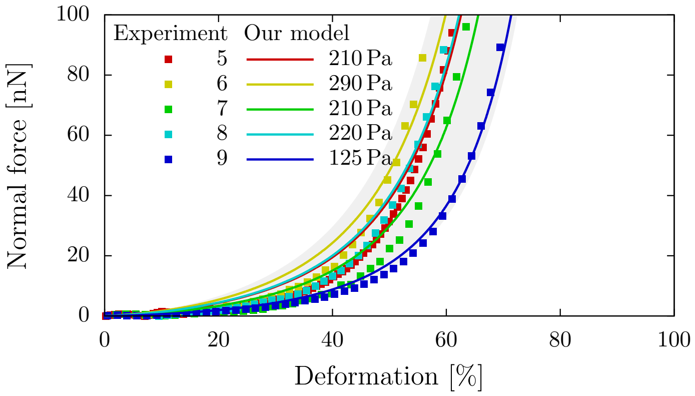

Fig. S-1 Our numerical model in comparison to our FluidFM® measurements on REF52 cells. The ratio of the shear moduli is chosen as $w = 0.25$ for all curves. The gray area shows the simulation of a cell with an averaged cell radius of $8.6(7)\,\mu\mathrm{m}$ and Young's modulus range $220 \pm 100\,\mathrm{Pa}$.

S-2 Supporting Information for the numerical model

S-2.1 Convergence of single cell deformation in shear flow

The temporal development of the deformation D of a single cell in a Couette flow can be seen in figure S-2. Starting from a spherical shape ($D = 0$), the cell experiences a shape change during an initial transient timespan, after which it assumes a steady shape. For capillary numbers $\mathrm{Ca} > 0.2$, we first find an overrelaxation of the deformation before it converges towards a constant value.

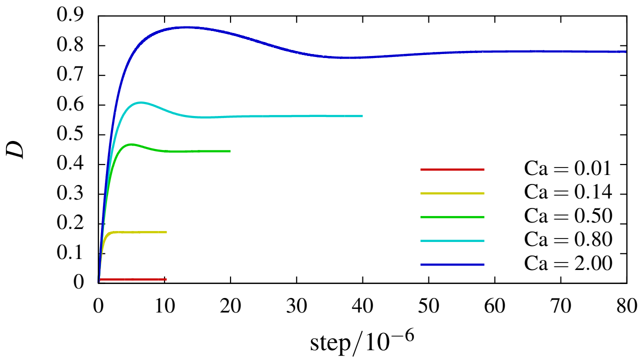

Fig. S-2 Single cell deformation in Couette flow for different capillary numbers. After an initial transient timespan, the deformation converges to a constant value.

S-2.2 Reduction of the system resolution

In figure S-3 we show that a system with reduced cell resolution (from $R_{\mathrm{Cell}} = 10$ to $R_{\mathrm{Cell}} = 6$ grid cells) and a smaller simulation box (from $100 \times 150 \times 100$ to $60 \times 90 \times 30$ grid cells) produces the same deformation versus capillary number behavior as the system with higher resolution.

S-2.3 Translational and rotational invariance of the force calculation

As a very direct test for the correct behavior of our model, we consider a single tetrahedron and examine the behavior of the volume and the elastic force for an initially applied translation, rotation and stretching. In figure S-4a, the behavior of the volume under these deformations is shown over the first time steps. While the volume remains constant under pure translation, pure rotation, and a combination of both, it quickly relaxes towards its reference value after an initial stretch is applied. The same behavior is observed for the elastic force acting on one tetrahedron vertex, in figure S-4b.

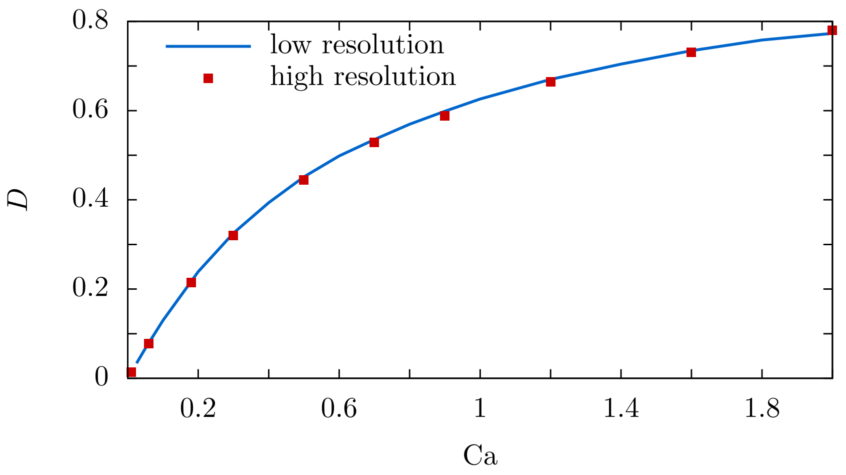

Fig. S-3 Taylor deformation as function of the capillary number for two different cell and channel resolutions. The large system ($R_{\mathrm{Cell}} = 10$, box: $100 \times 150 \times 100$ grid cells) produces the same outcome as the down-scaled system ($R_{\mathrm{Cell}} = 6$, box: $60 \times 90 \times 30$ grid cells).

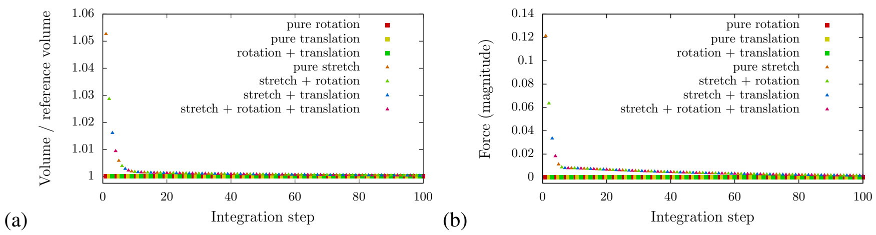

Fig. S-4 The behavior of (a) the volume and (b) the elastic force on a single vertex of a tetrahedron after an initial rotation, translation or stretching.

S-2.4 Mesh generation and mesh independence

The tetrahedral mesh of our spheroid is generated using the software gmsh (version 4.3.0) [1]. The Frontal2D meshing algorithm produced a mesh with highest uniformity considering edge length, triangle area and tetrahedron volume distribution. Nevertheless, all other available meshing algorithms produce likewise uniform meshes, with one exception being the Frontal3D algorithm, as listed in table S-2. We demand the uniformity of the mesh to increase the accuracy of our coupled Immersed-Boundary Lattice Boltzmann simulations. Figure S-5 shows the force-deformation curves for meshes with increasing number of tetrahedra, which are converged and thus prove sufficient sampling of the volume mesh.

Table S-2 Statistics of meshes created using different built-in algorithms of Gmsh [1]. Listed are edge length L, triangle area A, and tetrahedron volume V providing average, standard deviation, minimum and maximum value for each mesh.

Algorithm	Frontal2D	MeshAdapt	Delaunay2D	Delaunay3D	Frontal3D
$\bar{L}$	1.252	1.362	1.292	1.362	1.484
σ_L	0.243	0.301	0.299	0.301	0.530
L_{min}	0.616	0.588	0.592	0.588	0.510
L_{max}	2.138	2.345	2.462	2.345	3.622
$\bar{A}$	0.348	0.422	0.382	0.422	0.565
σ_A	0.377	0.473	0.436	0.473	0.837
A_{min}	0.218	0.228	0.192	0.228	0.204
A_{max}	1.577	1.851	1.709	1.851	4.444
$\bar{V}$	0.218	0.291	0.252	0.291	0.473
σ_V	0.078	0.121	0.112	0.121	0.405
V_{min}	0.049	0.051	0.043	0.051	0.049
V_{max}	0.600	0.881	0.840	0.881	2.353

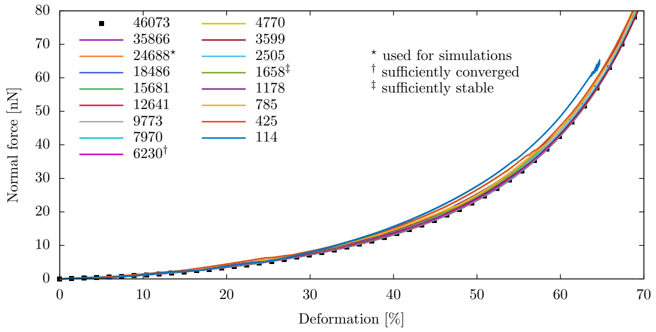

Fig. S-5 Force-deformation behavior of meshes with increasing number of tetrahedra. Meshes with $N \geq 1658$ tetrahedra are stable in the investigated range of deformation. Above 6230 tetrahedra, all meshes produce the same converged output. The following parameters were used: cell radius $R = 7.5\,\mu\text{m}$, Young's modulus $E = 300\,\text{Pa}$, and Poisson ratio $\nu = 0.48$.

S-2.5 Hertz theory

Although originally designed for the contact between two linear elastic spheres, the Hertz theory can be applied to the contact between a linear elastic sphere and a flat plate [2]. The general assumptions for the Hertz-theory are the following [3, p. 91-92]:

- frictionless, smooth contact surfaces
- contact area small compared to sphere dimension
- homogeneous, isotropic and linear elastic material

S-2.5.1 Sphere-sphere contact

The following quantities are necessary to describe the normal contact of two elastic spheres. The radii R_1 and R_2 of the spheres define the effective radius of curvature R

of the bodies by

$$\frac{1}{R} = \frac{1}{R_1} + \frac{1}{R_2} \quad . \tag{S-1}$$

Through their Young's moduli and the Poisson ratios, E_1, E_2 and ν_1, ν_2, the effective stiffness K is defined as:

$$\frac{1}{K} = \frac{1-\nu_1^2}{E_1} + \frac{1-\nu_2^2}{E_2} \tag{S-2}$$

The displacement δ, which measures the distance that the sphere centers approach each other due to a normal force N acting on each sphere, can be expressed in terms of the above parameters [2]:

$$\delta = \left(\frac{9N^2}{16RK^2}\right)^{\frac{1}{3}} \tag{S-3}$$

Therefore, the force–displacement relation according to the Hertzian theory for a sphere-sphere contact is given by

$$N(\delta) = \frac{4}{3} K R^{\frac{1}{2}} \delta^{\frac{3}{2}} \quad . \tag{S-4}$$

S-2.5.2 Sphere-plane contact

The analytical solution for the force–displacement relation according to the Hertzian theory for the contact of a linear elastic sphere with a rigid plane can be obtained from (S-4) by applying the following modifications: the plane has no curvature, thus $R_2 \to \infty$ and (S-1) simply yields $R = R_1$. Since the plane is assumed rigid, i. e. $E_2 \gg E_1$, (S-2) reduces to $K = \frac{E_1}{1-\nu_1^2}$. In this case, N is the force acting on the sphere and δ is the distance between the center of the sphere and the plane.

S-2.6 Influence of the Mooney-Rivlin ratio w

To clarify the influence of w, we plot in figure S-6 the force versus deformation behavior of our cell model for different values of w. With decreasing w, i. e. decreasing μ_1 while increasing μ_2, the strain hardening effect clearly increases and the upturn of the force curve begins at lower deformations. This is due to μ_2 scaling the term in the strain energy density that is quadratic with the deformation (cf. equations (4) and (5) of the manuscript).

S-2.7 Influence of the Poisson ratio ν

In figure S-7 we demonstrate that variations of the Poisson ratio ν within the range of an approximately incompressible material do not notably influence the force-deformation curves.

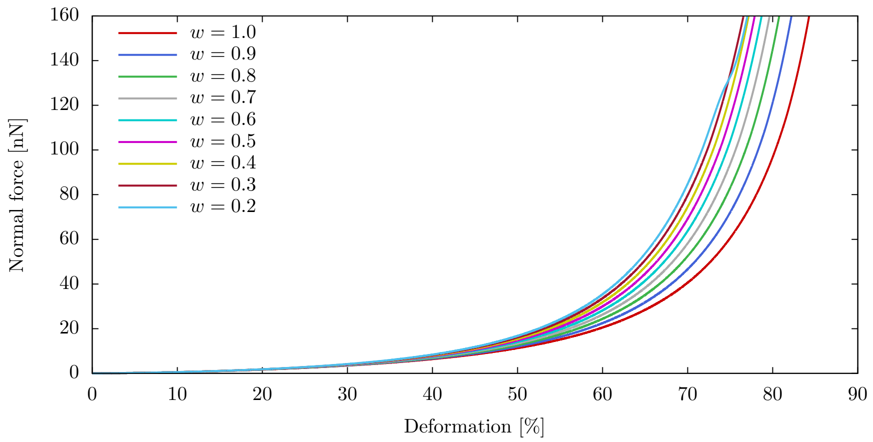

Fig. S-6 Variation of w: The lower w (the lower μ_1 compared to μ_2), the stronger the non-linear upturn of the force becomes. The curve with $w = 1$ corresponds to the one in Fig. 4 of the manuscript with a Young's modulus of $E = 160\,\mathrm{Pa}$.

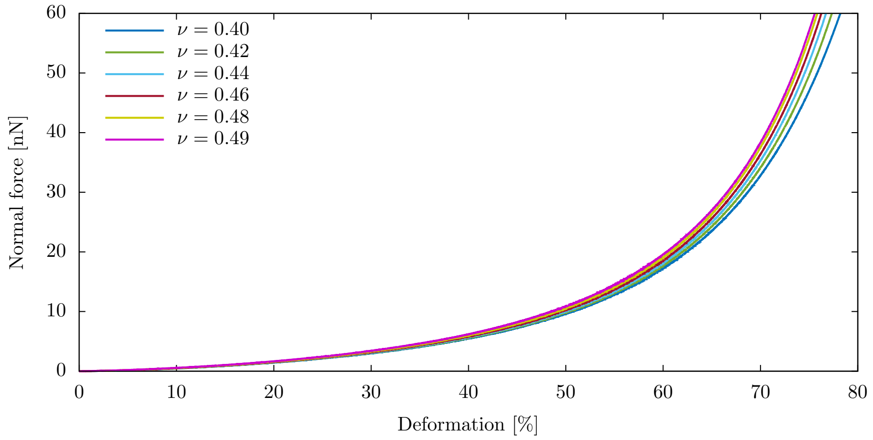

Fig. S-7 Force versus deformation curves for different Poisson ratios ν. The following parameters were used: cell radius $R = 7.5\,\mu\mathrm{m}$ and Young's modulus $E = 300\,\mathrm{Pa}$.

S-3 Compression and indentation simulations

After initialization, each time step of our overdamped relaxation simulation consists of the following two steps: the movement of the upper wall to compress – or the sphere to indent – the cell and the integration of the equation of motion of the cell vertices,

$$\dot{\mathbf{y}}^{\alpha} = \gamma^{-1}(\mathbf{f}^{\alpha} + \mathbf{f}^{\alpha}_{\text{probe}}) \quad . \tag{S-5}$$

The vertex velocity $\dot{\mathbf{y}}^{\alpha}$ is obtained from the elastic restoring forces ($\mathbf{f}^{\alpha}$ (12) and the probe repulsion $\mathbf{f}^{\alpha}_{\text{probe}}$), considering a friction factor γ. Since here we are only looking at a sequence of equilibrium states, the value of γ is irrelevant for the resulting force-deformation curves and only influences the performance and stability of the simulations. The equation of motion is integrated using a fourth order Runge-Kutta algorithm. The repulsive cell-probe interaction, preventing the cell vertices from penetrating the plates or the indenter, has the form

$$\mathbf{f}_{\text{probe}}(d) = \frac{c_{\text{F}}}{d^2}\mathbf{n} \quad , \tag{S-6}$$

with the cell-probe distance d and a proportionality factor c_{F}. The force points normal to the probe, resulting in a compression between two plates and a radial displacement away from the indenter. Physically, this corresponds to a free-slip boundary condition which does not restrict tangential motions of the cell along the probe.

S-4 Flow simulations with Lattice Boltzmann

S-4.1 Method

This section briefly summarizes the Lattice Boltzmann method implemented in the open-source package ESPResSo [4]. For an introduction into the Lattice Boltzmann method we refer the interested reader to the book by Krüger et al. [5]. The Lattice Boltzmann equation for the multiple relaxation time scheme used in ESPResSo reads:

$$f_i(\mathbf{x}+\mathbf{c}_i\Delta t, t+\Delta t) - f_i(\mathbf{x},t) = \sum_{j=0}^{18} \left(\mathsf{M}^{-1}\omega\,\mathsf{M}\right)_{ij} \left(f_j(\mathbf{x},t) - f_j^{\mathrm{eq}}(\mathbf{x},t)\right) \qquad \text{(S-7)}$$

It describes the collision and streaming of the population distribution f_i ($i = 0,\dots,18$) during one time step Δt. Here, $\mathbf{c}_i$ are the discretized lattice velocities, M denotes transformation matrix that maps the populations onto moment space, ω is the diagonal relaxation frequency matrix, and f_i^{eq} denote the equilibrium population distributions. The relaxation frequency for the shear moments ω_{S} is related to the dynamic viscosity of the fluid via [6]

$$\eta = \rho c_s^2 \left(\frac{1}{\omega_{\mathrm{S}}} - \frac{1}{2}\right)\Delta t \quad , \qquad \text{(S-8)}$$

with the fluid mass density ρ and the lattice speed of sound c_s. In order to ensure simulation stability, we choose the time step globally according to Krüger *et al.*[5, p. 273] as

$$\Delta t = c_s^2\left(\tau - \tfrac{1}{2}\right)\frac{\Delta x^2}{\nu}\tilde{t} = \frac{\Delta x^2}{6\nu}\tilde{t} \quad , \qquad \text{(S-9)}$$

with $c_s^2 = \frac{1}{3}$, a global relaxation parameter $\tau = 1$, the kinematic viscosity ν, and an additional factor $\tilde{t}$ in the range 1–2 to manually tune the time step.
We further introduce a scaling factor r by which we divide both the viscosity and the Young's modulus. According to eq. (S-9), this leads to a larger time step and thus to a speed-up of the simulations. At the same time it leaves the important Capillary number unchanged and only increases the Reynolds number, which nevertheless remains $\ll 1$. The parameter r thus does not affect the physics of the simulation which we have carefully checked by a number of test runs with $r = 1$.
At the boundaries of the channel a bounce-back algorithm is applied to realize a no-slip boundary condition. For the plane Couette setup, the bounce-back algorithm additionally allows for a fixed tangential velocity component.
We use a combined CPU/GPU implementation which enables the calculation of the flow field on the GPU, while the calculation of the cell motion is done in parallel on multiple (4 to 20) CPUs. In lattice units, our simulation box for the single cell in shear flow setup (cf. section 6.1) has the dimensions $60 \times 90 \times 30$ ($x \times y \times z$), for the multiple cell simulation (cf. section 6.2) it is $50 \times 80 \times 40$. The dynamic viscosity, chosen as $\nu = 1$ in simulation units, determines the time step in our simulations as $\Delta t = \frac{1}{3}$ with $\tilde{t} = 2$.

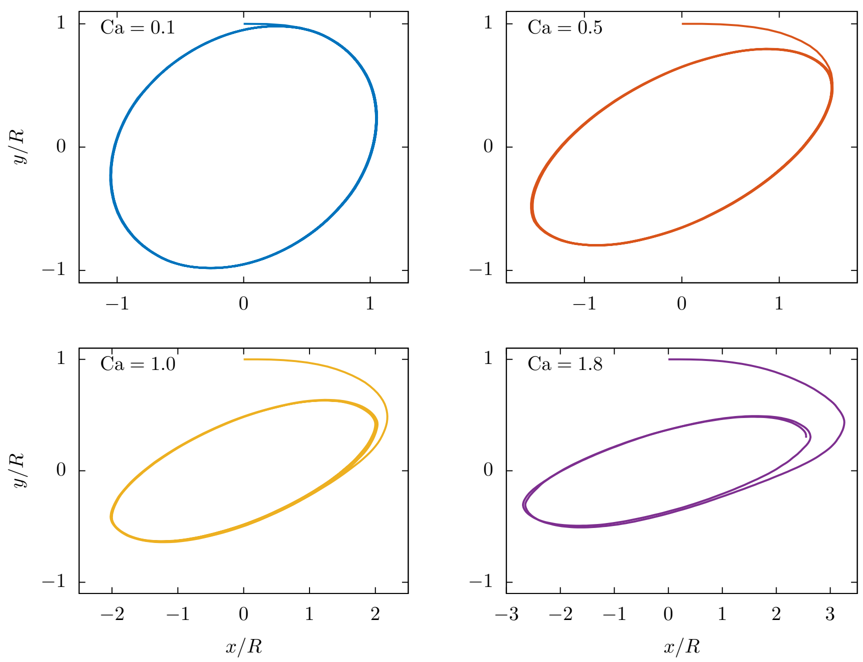

Fig. S-8 The trajectory of a surface node (here: starting at $y = R$ and $x, z = 0$) for different capillary numbers traces the ellipsoidal contour of the deformed particle. The non-elliptical part of the trajectory in the upper-right corner represents the approach from the initially spherical to the final shape.

S-4.2 Tank-treading motion

Figure S-8 shows the trajectories of selected vertices on the outer surface of the particle for different capillary numbers. They describe an ellipsoidal motion tracing the outer contour of the deformed particle thus demonstrating that in our simulations the particle exhibits tank-treading.

References

1. C. Geuzaine, J.F. Remacle, International Journal for Numerical Methods in Engineering **79**(11), 1309 (2009). DOI 10.1002/nme.2579
2. E. Dintwa, E. Tijskens, H. Ramon, Granular Matter **10**(3), 209 (2008). DOI 10.1007/s10035-007-0078-7
3. K.L. Johnson, *Contact Mechanics*, 9th edn. (Cambridge Univ. Press, Cambridge, 2003). OCLC: 250004367
4. H. Limbach, A. Arnold, B. Mann, C. Holm, Computer Physics Communications **174**(9), 704 (2006). DOI 10.1016/j.cpc.2005.10.005
5. T. Krüger, H. Kusumaatmaja, A. Kuzmin, O. Shardt, G. Silva, E.M. Viggen, *The Lattice Boltzmann Method*. Graduate Texts in Physics (Springer International Publishing, Cham, 2017). DOI 10.1007/978-3-319-44649-3

6. Z. Chai, B. Shi, Z. Guo, F. Rong, Journal of Non-Newtonian Fluid Mechanics **166**(5-6), 332 (2011). DOI 10.1016/j.jnnfm.2011.01.002
